# Developing a Commercial Antimicrobial Active Packaging System of Ground Beef Based on “*Tsipouro*” Alcoholic Distillate

**DOI:** 10.3390/foods9091171

**Published:** 2020-08-25

**Authors:** Anastasia E. Kapetanakou, Georgia-Lito Pateraki, Panagiotis N. Skandamis

**Affiliations:** Laboratory of Food Quality Control and Hygiene, Department of Food Science & Human Nutrition, Agricultural University of Athens, Iera Odos 75, 11855 Athens, Greece; akapet@aua.gr (A.E.K.); lito_pater@msn.com (G.-L.P.)

**Keywords:** ground beef, active packaging, alcoholic distillate, ethanol, predictive modelling, shelf-life

## Abstract

The present study aimed to develop a commercial active packaging system of ground beef, by exploiting the antimicrobial and antioxidant properties of a traditional Greek alcoholic distillate called “*tsipouro*”. Commercial packages (500 g) were used and 40 mL of “*tsipouro*” was added in absorbent pads placed underneath the ground beef, while 10 mL was also mounted under the packaging film, facing the headspace. Samples were packaged in 80% O_2_: 20% CO_2_ and stored at 0, 4, 8, and 12 °C. Total Viable Counts, pseudomonads, *Brochothrix thermosphacta*, lactic acid bacteria, yeasts-moulds, pH, colour (L*, a*, b*), odour (buttery and acidic), and ethanol migration to ground beef (SPME/GC-FID) were determined. Moreover, mathematical models (square root and Arrhenius) describing the effect of temperature on determinant indicators of spoilage and quality deterioration like growth of dominant microorganisms and red colour reduction were developed and validated under non-isothermal conditions. *B. thermosphacta* dominated the microbial association of ground beef, while LAB were second in dominance, revealing a high growth potential at all assays. a* value (redness) was gradually decreased in controls, while samples treated with “*tsipouro*” showed more stable red colour during storage. Although ethanol was organoleptically detectable, especially at low storage temperatures (0–4 °C), it was rather perceived as a pleasant cool odour. Prediction by both models for microbial growth as well as those of Arrhenius model for reduction of a* value showed good agreement with the observations under non-isothermal storage. Overall, our study showed that the developed antimicrobial active packaging of ground beef based on “*tsipouro*”, combined with high oxygen MAP lead to an almost 2-fold shelf-life extension compared with controls during storage at chill and abuse temperatures.

## 1. Introduction

Beef meat is considered a popular food commodity, possessing the third place in per capita consumption among all meats worldwide, although is excluded from the nutrition of some cultures either for religious or financial reasons, as being more expensive than pork or chicken meats [1,2]. According to recent report of Grand View Research Inc. (2019), the global beef meat market size is expected to reach USD 383.5 billion by 2025, exhibiting a 3.1% compound annual growth rate (CAGR) during the forecast period of 2019–2025 [3]. Given that 64% of beef meat is consumed as ground beef [4] along with its high perishability as a main drawback, its shelf-life extension constitutes a major challenge for the meat industry. It is well-known that the quality of ground beef deteriorates during storage and distribution predominantly due to microbial changes, discoloration, and lipids or proteins oxidation [5]. Until now, meat industry overcomes these undesirable alterations by applying modified atmosphere packaging (MAP) of 80% O_2_: 20% CO_2_, resulting to final products with attractive red meat colour and a shelf-life of ca. 10 days at refrigerated temperatures, thus allowing distribution of a consistent and cost-effective product to retail [6]. However, some defects may also arise, when high oxygen MAP is applied, such as rancidity, off-flavours, pre-matured browning, and tenderness [7,8].

Given the aforementioned limitations of MAP, complementary preservation technologies (“hurdle effect”) could be deployed [9]. Active packaging is a promising packaging technique that may successfully serve this additional role. This technique goes beyond the traditional packaging functions, since the package, the product, and its environment interact to improve safety, quality, and sensory properties of the packaged food [10]. Among its versatile applications, active packaging via the emission of antimicrobial volatile substances, i.e., ethanol, essential oils, and plant extracts has stimulated scientific interest, since they may actively modify the atmosphere inside the food package throughout storage and distribution [11,12,13]. With regards to ethanol, its antifungal and antibacterial properties are well-documented. Specifically, from the aspect of the food industry, spraying of ethanol prior to packaging of, i.e., bakery products, is a common practice; however, generating ethanol by adhesive-backed films or sachets is also applied. In fact, many applications in the form of sachets have been patented and launched in the market mainly by Japanese manufacturers i.e., Antimold Mild^TM^, Antimold Tender^TM^ (Freund, Japan), Oitech^TM^ (Nippon Kayaku Co. Ltd.). Although ethanol-emitting packaging is stipulated by 2011/10/EC Regulation, the addition of an article inside the food package may sometimes cause consumers to hesitate [14]. Another issue that may reduce purchase willingness is the acceptance of pure ethanol application in food. Alternatively to ethanol, alcoholic beverages could be used due to their content in alcohol and phenolic compounds, especially for products which are intended to be cooked prior to consumption like ground beef. In fact, several international cuisines have adopted the addition of wines, spirits, and liquors during cooking in order to add flavour to food. In Greek cuisine, wine along with the traditional, alcoholic beverages “*tsipouro*”, “*raki*”, or “*ouzo*” are among the most popular used. “*Tsipouro*” and “*raki*” are produced by single and double distillation of grape pomace, respectively, while “*ouzo*” is being produced by infusing pure white alcohol, after double or triple distillation process, with various herbs such as aniseed, liquorice, and mint. Taking the latter into account and in an effort to exploit the antimicrobial and antioxidant properties of alcoholic beverages starting from products’ packaging and during distribution, our research group has previously tested the vapours of distillery ethanol or different alcoholic beverages (international like brandy or whiskey vs. Greek, traditional like “*tsipouro*”, “*ouzo*”, and “*raki*”) on pork meat combined with MAP, resulting in a shelf-life extension by almost 2-fold compared to controls during storage at 4 °C [15]. The antimicrobial effect of the aforementioned Greek alcoholic beverages was also verified against the pathogen *Listeria monocytogenes* when were applied via edible films on frankfurters and ham slices, resulting in a 4.0–5.0 log CFU/cm^2^ inhibition compared to controls during storage at 4 and 10 °C [16]. Although, such applications are based on the hypothesis that ethanol content is significantly evaporated after cooking, additional care should be taken with industrial products containing ethanol or meals cooked with alcoholic drinks in case of consumption by the children, abstinent alcoholics, or ethanol-sensible individuals, since according to previous studies the higher the initial amount of ethanol added, the greater its final concentration was after cooking [17].

Considering that the positive antimicrobial effect of the aforementioned Greek alcoholic beverages tested previously by our research group has been recorded in samples of laboratory scale, the main idea of the present study was to set an extra challenge by developing a commercial antimicrobial active packaging system of ground beef, exploiting also the already used pads, placed underneath the product for the absorption of meat liquids excess, as potential carriers of Greek “*tsipouro*”, which is already used by the consumers during cooking. In order to evaluate the efficacy of the developed antimicrobial active packaging system and strengthen its potential application by the meat industry, the followed objectives were set: (i) The evaluation of microbial, quality, and sensory attributes of ground beef during different storage temperatures, (ii) the fitting of produced data to mathematical models describing the effect of temperature on determinant indicators of spoilage and quality deterioration like growth of dominant microorganisms and red colour reduction, and (iii) the validation of the developed models under non-isothermal conditions.

## 2. Materials and Methods

### 2.1. Antimicrobial Compound

“*Tsipouro*” is a traditional grape marc distillate produced in continental Greece, i.e., Thessaly, Epirus, Macedonia, or the island of Crete (known as “*tsikoudia*” or “*raki*”) from residues of the winemaking process of several grape varieties [18,19]. It could be found in two forms, either pure or anise-flavoured. “*Tsipouro*” production process is summarized in: (i) Fermentation of the grape marc followed by two sequential distillations, (ii) collection of the second distillate and (iii) dilution with water to a final ethanol content of 37.5–45% (*v*/*v*) [20]. In the present study, a commercially-available “*tsipouro*” of 38% *v*/*v* alcohol content without anise was used in order to create the studied antimicrobial active packaging.

### 2.2. Preparation and Storage of Ground Beef

**Figure 1:**
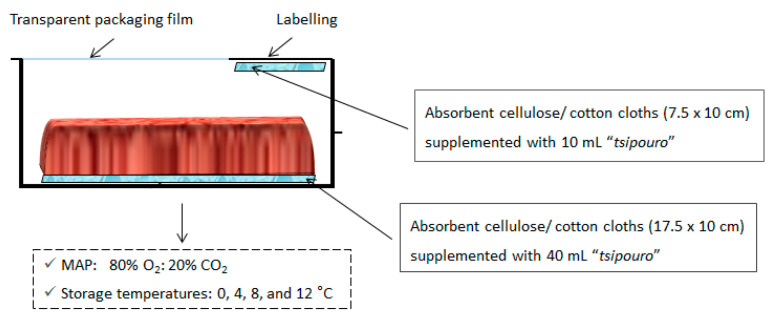
Proposed antimicrobial active packaging of ground beef (500 g) by applying “*tsipouro*” under modified atmosphere packaging (MAP) of 80% O_2_: 20% CO_2_.

Freshly prepared commercial packages of ground beef (500 g; 81% lean: 19% fat; pH 5.54 ± 0.05) packaged under modified atmospheres (MAP) 80% O_2_: 20% CO_2_ were kindly provided by a leading Greek meat industry (Athens, Greece) and were transported to the laboratory within 30 min, where they were stored at 2–4 °C for 1 h. The packages were opened and the ground beef piece (500 g; 15.5 × 8.5 cm) was aseptically removed out of the tray and placed on a sterile surface. The (pre-) existing absorbent pad at the bottom of each package tray was replaced by new one (17.5 × 10 cm; cellulose/cotton; provided by the meat industry) and aliquots of 40 mL of “*tsipouro*” were added. Afterwards, the whole ground beef piece was placed back to the package on top of the moistened absorbent pad. All trays were placed in plastic bags (gas permeability rate ca. 25, 90, and 6 cm^3^/m^2^/day/10^5^ Pa for CO_2_, O_2_, and N_2_, respectively at 20 °C) (data provided by the manufacturer; Flexo-Pack S.A., Athens, Greece) and an additional absorbent pad of similar material (7.5 × 10 cm) supplemented with 10 mL of “*tsipouro*” was also mounted under the packaging film of each package, facing the headspace (Figure 1). The present set-up resulted in “*tsipouro*” migration via diffusion through the contact area between absorbent pad and ground beefs’ bottom side and through evaporation by the pad placed on the top. All trays were re-packaged under 80% O_2_: 20% CO_2_ using a gas flush sealing packaging machine (Henkovac 1900 Machine, ‘s-Hertogenbosch, The Netherlands) and stored at 0, 4, 8, and 12 °C in high precision (±0.5 °C) incubation chambers (MIR-153, Sanyo Electric Co., Osaka, Japan). The pads served as vehicles (“carriers”) of the antimicrobial, allowing the gradual release of “*tsipouro*” vapours in the packaging atmosphere during storage. Samples packaged under the same conditions (MAP, packaging film, and storage temperatures) but without applying “*tsipouro*” served as controls.

### 2.3. Headspace Gas Analysis

Headspace atmosphere composition in each package of ground beef was monitored using a portable PBI Dansensor A/S (Check Mate 9900 O_2_/CO_2_; Ringsted, Denmark) analyser (accuracy ± 0.1%) with oxygen and carbon dioxide sensors. Three (3) mL of gas from the package headspace were sampled automatically by the gas analyser with a medical type needle. The needle was inserted in the headspace via a septum glued on the external part of the MAP pouch. Septum was used to prevent crack propagation in the film package due to the puncture.

### 2.4. Microbiological Analysis

Ground beef samples (10 g) were aseptically taken by sampling transversely sections from the packaged ground beef, added to 90 mL of sterile ¼ strength Ringer’s solution (Lab M, Lancashire, UK), and homogenized in a stomacher (Interscience, Paris, France) for 60 s. Following homogenization, decimal dilutions in ¼ strength Ringer’s solution were prepared and 1 or 0.1 mL of the appropriate dilutions were poured or spread respectively, on selective and non-selective culture media. Total Viable Counts (TVC) were enumerated on Plate Count Agar (PCA; Lab M, Lancashire, UK) after incubation at 30 °C for 48 h; lactic acid bacteria (LAB) on double layer of De Man, Rogosa and Sharp agar (MRS; Lab Lancashire, UK) (pH 5.8) at 30 °C for 72 h; *Pseudomonas* sp. on Pseudomonas Selective Agar (Lab M, Lancashire, UK) with Cetrimide–Fucidin–Cephaloridine supplement (CFC; Lab M, Lancashire, UK) agar incubated at 25 °C for 48 h; *B. thermosphacta* on STAA Agar Baser (Biolife Italiana Srl, Monza MI, Italy) with STAA Selective Supplement (Biolife Italiana Srl, Monza MI, Italy) incubated at 25 °C for 48 h; yeasts and moulds on Rose Bengal Chloramphenicol Agar (RBC; Lab M, Lancashire, UK) incubated at 25 °C for 96 h; Enterobacteriaceae on a double layer of Violet Red Bile Glucose Agar (VRBGA; Lab M, Lancashire, UK) incubated at 37 °C for 24 h.

The microbial shelf-life of ground beef was assessed by the following equation [21]:

(1) $$SL_{microbial}=\;t_{lag}\;+\;(\ln10)\times \frac{\log N_{s}\;-\;\log N_{0}}{\mu_{max}}$$

where, *SL_microbial_* is the estimated shelf-life (day) based on the microbiological data of *B. thermosphacta* which was the specific spoilage organism, *t_lag_* is the lag time (day), *µ_max_* the maximum specific growth rate (day^−1^), *N*_0_ is the initial enumerated population (log CFU/g), and *N_s_* the minimal spoilage level set at 7.0 log CFU/g according to Ercolini et al. (2011) [22]. Parameters of *t_lag_* and *µ_max_* were calculated by the Baranyi model as described below in Section 2.6.

### 2.5. PH Measurement

The pH values were recorded at every sampling with a digital pH meter (pH 526, Metrohm Ltd., Herisau, Switzerland) by immersing the glass electrode in the homogenate of ground beef following microbiological analysis.

### 2.6. Model Development of B. thermosphacta and LAB Growth

*B. thermosphacta* and LAB were selected to be modelled due to their dominance in the present experimental set up. Specifically, a typical two-stage modelling approach was applied. Primary modelling was carried out using the Baranyi growth model via DMFit (available at http://www.combase.cc/index.php/en/) [23]. Growth curves of *B. thermosphacta* and LAB were generated by plotting bacterial population (log CFU/g) vs. storage time, while maximum specific growth rate (*µ_max_*; day^−1^), lag time (*λ*; day), initial microbial load (*N*_0_; log CFU/g) and maximum microbial load (*N_m_*_ax_; log CFU/g) were estimated per storage temperature for controls and samples treated with “*tsipouro*”.

Maximum specific growth rates (*µ_max_*) of *B. thermosphacta* and LAB for controls and “*tsipouro*” samples were secondarily modelled as a function of storage temperature using square root model (Equation (2)) [24] and Arrhenius equation (Equation (3)) [25]:

(2) $$\sqrt{\mu_{max}}=b\;\left(\;T\;-\;T_{0}\right)$$

where *b* is a constant, *T* (°C) is the storage temperature, and *T*_0_ is the notational (theoretical) minimum temperature for growth of *B. thermosphacta* and LAB, estimated by extrapolation of the regression line to *µ_max_* = 0. The parameters of the developed square root models were calculated using the IPMP (Integrated Pathogen Modeling Program, USDA, Pennsylvania) software [26].

(3) $$\ln\mu_{max}=\;\ln(\mu_{ref})\;-\;\left(\frac{E_{a}}{R}\right)\left[\frac{1}{T}\;-\;\frac{1}{T_{ref}}\right]$$

where, *µ_ref_* (day^−1^) is the maximum specific growth rate of *B. thermosphacta* and LAB at the reference temperature *T_ref_* (K), which was set at 4 °C, *T* is the absolute temperature (K), *E_a_* is the activation energy (J/mol) and *R* is the universal gas constant (J/mol·K). *E_a_* values were estimated from the slope of Arrhenius plots of ln (*k*) vs. (1/T − 1/T_ref_) by linear regression [27]. All model parameters were calculated using SPSS computer package Version 16.0 (SPSS Inc., Chicago, IL, USA).

### 2.7. Colour Measurement

Following sampling for microbiological analysis, ground beef pieces were immediately subjected to colour measurements. The changes in colour were evaluated by measuring the L*, a*, b* parameters (CIELab scale) using a portable colorimeter (Minolta, Model CR-200, Osaka, Japan) on the top and bottom surface of ground beef, as configured in the package. Specifically, L* value (lightness) is a measure of total light reflected on a scale ranging from 0 = black to 100 = white; a* value (redness) is a measure of the red (positive values) and green (negative values) colours, while b* value (yellowness) is a measure of the yellow (positive values) and blue (negative values) colours of the sample. The colorimeter was calibrated as described in the user’s manual using a white standard plate (*L* = 100). After calibration, colour was measured by taking ten readings in a total area of 25 cm^2^ per treatment (controls and “*tsipouro*” samples) and sampling side (surface and bottom).

### 2.8. Model Development of a* Value

Considering that in red meat a* value (redness) is the most critical determinant for consumer acceptance, it was also selected for modelling purposes. Thus, a* value was plotted vs. storage time and the apparent rate of colour deterioration *k_colour_* (day^−1^) was estimated per storage temperature, sampling side (surface and bottom) and treatment (controls and “*tsipouro*”) accordingly to the linear equation below:

a* = a_0_* + *k_colour_ t* (4)

where, a* is the a* value after storage time t (day), *a*_0_* is the initial value at time zero.

The rate of colour deterioration (*k_colour_*) of ground beef was further modelled as a function of storage temperature using the Arrhenius equation (Equation (5)) [25]:

(5) $$\ln(k_{colour})=\;\ln(k_{ref})\;-\;\left(\frac{E_{a}}{R}\right)\left[\frac{1}{T}\;-\;\frac{1}{T_{ref}}\right]$$

where, *k_ref_* (day^−1^) is the constant rate of the degradation of a* value at the reference temperature *T_ref_* (K), which was set at 4 °C, *T* is the absolute temperature (K), *E_a_* is the activation energy (J/mol) and *R* is the universal gas constant (J/mol·K). *E_a_* values were estimated from the slope of Arrhenius plots of ln (*k_colour_*) vs. (1/T − 1/T_ref_) by linear regression [27]. All model parameters were calculated using SPSS computer package Version 16.0 (SPSS Inc., Chicago, IL, USA).

### 2.9. Validation of the Developed Models for Microbial Growth and Colour Deterioration

Validation experiments were designed in order to assess the efficiency of the fitted models in predicting microbial growth and colour deterioration of ground beef, under dynamic storage temperatures ranging from 0 to 12 °C. Storage of packages took place in high precision (±0.5 °C) incubation chambers (MIR-153, Sanyo Electric Co., Osaka, Japan). Temperature fluctuations were recorded by a computer downloadable electronic data logger (testo 174, Testo Inc., NJ, USA) placed inside the incubators. Changes in microbiological parameters as well as in colour were monitored as described in Section 2.4 and Section 2.7, respectively.

The predictions of microbial growth and a* value changes were based on the time-temperature profiles obtained by the data loggers and the secondary models, i.e., the square root (Equation (2)) and Arrhenius equations (Equations (3) and (5)) for the estimation of the ‘‘momentary’’ *µ_max_* and *k_colour_* between two consecutive reads. The derived *µ_max_* was then introduced into the Baranyi model for certain *N*_0_ and *h*_0_ to simulate the microbial growth of *B. thermosphacta* and LAB, packaged with or without “*tsipouro*” and under non-isothermal storage temperatures. With regards to a* value, the derived *k_colour_* was introduced into the primary linear model (Equation (4)), for the estimated (fixed) ln *k_ref_* and *E_a_* values. The initial concentration (*N*_0_; log CFU/g) of microbial growth and the initial *a*_0_* value for colour, were used as the starting point of simulation as determined by the plate count method (Section 2.4) or the colorimetric method (Section 2.7), respectively. Regarding the lag time of the Baranyi growth model, *h*_0_ is the parameter characterizing the “adaptation work” needed by the cells to enter the exponential phase or ‘‘relative lag’’ and is the product of *µ_max_* and lag [28]. For the simulation, *h*_0_ was fixed arbitrary to a low value of 0.1 (i.e., practically indicating absence of lag time), since no lag was determined in most of the studied storage temperatures for both spoilage microorganisms.

### 2.10. Evaluation of Models Performance

The following statistical indicators were used to evaluate the performance of the models: (i) Regression coefficient (R^2^) and (ii) residual mean square error (RMSE) of each model. The higher the R^2^ value and the lower the RMSE value, the better was the goodness-of-fit of the model.

The agreement between predicted and observed populations of *B. thermosphacta* and LAB as well as a* value was assessed through the accuracy (A*f*) (Equation (6)) and bias (B*f*) factors (Equation (7)) [29]. Specifically, A*f* and B*f* were calculated by taking into account the fitted and observed data (log CFU/g of *B. thermosphacta* and LAB; a* value) per studied assay.

(6) Af=10^[∑1n|log(DatafittedDataobserved)|n] 

(7) Bf=10^[∑1nlog (DatafittedDataobserved)n]

where *n* equals to the number of observations. Perfect agreement between fitted and observed values is represented with accuracy and bias factors of 1 [29]. *Bf* values of 1.00–1.15 indicate that predictions exceed observations (over-prediction), while values between 0.70 and 1.00 are interpreted as under-prediction [30].

Likewise, relative error (RE) of *B. thermosphacta* and LAB populations (log CFU/g) or a* value of individual predictions were calculated [31] by the following equation:

RE = (predicted − observed)/predicted (8)

and RE < 0 represented under-prediction and RE > 0 represented over-prediction of the developed models. The proportion of RE (pRE) that fell in an acceptable prediction zone (namely the number of RE in the acceptable prediction zone/total number of prediction cases) from an RE of −0.3 (under-predictions) to 0.15 (over-predictions) was calculated and used also as a measure of model performance. Models with pRE ≥ 0.70 were considered to provide predictions with acceptable bias and accuracy [32].

### 2.11. Sensory Evaluation

Sensory evaluation was performed during storage of extra samples in the expected commercial size of package. The same individuals participated in each evaluation, which was performed at the same time intervals as the rest studied parameters. Sensory evaluation was performed by 10 individuals (7 females and 3 males aged 25–40 years) under artificial light and at ambient temperature without knowing samples identity. All panellists were laboratory staff, a fact that enhances the reliability of sensory evaluation, since they were all familiar and experienced with sensory descriptors relevant to the process of meat spoilage; however, none was closely associated with the present study. Extra samples kept frozen and thawed prior to each sensory evaluation, were considered as fresh. Each evaluation was carried out twice and two samples per trial were tested (*n* = 4). The panellists were asked to assess the intensity of buttery (cheesy) or acidic (sour) odour, since they well-related to *B. thermosphacta* and LAB (dominant microbiota) growth, respectively [33]. Sensory evaluation was performed on a five-point hedonic scale from 1 to 3 with 0.5 intervals (1 = no buttery or acidic odour, 1.5 = slightly buttery or acidic odour, 2.0 = small buttery or acidic odour, 2.5 = moderate buttery or acidic odour, and 3 = extreme buttery or acidic odour). A score of 2.0 was the first indication that a sample changes from typical fresh to deteriorated meat however the sample was considered still acceptable by the panellists. Samples scored > 2 were considered as spoiled and indicated the end of the shelf-life. Moreover, the panellists were also asked to qualitatively evaluate the presence of ethanol by reporting its positive or negative perception (yes/no answer).

### 2.12. SPME/GC-Flame Ionization Detector Analysis

The main purpose of the present section was to monitor ethanol migration through diffusion and/or evaporation in the ground beef during storage, when “*tsipouro*” was applied compared to the metabolically produced ethanol on controls. Specifically, ethanol in ground beef was isolated by the headspace solid phase micro-extraction method (SPME). The fibre used for ethanol absorption was a DVB/CAR/PDMS—50/30 μm (Sigma-Aldrich, MO, USA). Samplings were performed from the top surface and the bottom side of ground beef at the beginning (day 0), the first sampling day, in the middle of storage (i.e., the day that TVC reached close to their maximum population level), and at the end of storage per temperature. Considering that diffusion of ethanol will be reduced from the outside to the centre of the ground beef piece (500 g), as a representative sample was decided to be a layer across the top surface or the bottom of the ground beef piece by removing 1 cm in depth. Each portion was mixed and 1 g was added along with the addition of 10 mL water into a 20 mL vial hermetically closed using a mininert valve (Sigma-Aldrich). Each vial was placed in a water bath for 15 min at 30 °C, while, subsequently, the fibre was exposed to the headspace of the vial for another 15 min at the same temperature. The fibre was then placed in the injection port of a gas chromatograph (Hewlett-Packard 5890 Series II, CA, USA) equipped with Flame Ionization Detector (Hewlett-Packard, CA, USA). Thermal desorption of ethanol from the fibre inside the GC injection port was carried out at 220 °C for 7 min. Gas chromatograph was equipped with an D8-WAX capillary column (30 m × 0.32 mm × 0.25 µm), and the carrier gas used was helium (1 mL min^−1^). For the analysis of ethanol the GC oven temperature was programmed from 40 °C (held for 5 min) to 150 °C at 12 °C/min and a second increase to 250 °C (held for 12 min) at 15 °C/min, resulting in a time-temperature program of 32.9 min. Ethanol in controls and “*tsipouro*” samples was semi-quantified by producing a calibration curve in the range of 0–1000 μL/L of ethanol under the same SPME conditions applied during the analysis of ground beef samples.

### 2.13. Statistical Analysis

Two independent storage experiments were performed and duplicate samples were used for each trial (*n* = 4). Statistical analysis was performed using the SPSS computer package Version 16.0 (SPSS Inc., Chicago, IL, USA). Analysis of variance (ANOVA) was performed on data to evaluate the effect of storage temperature and treatment on gas composition, microbial levels, pH, colour, ethanol content, and sensory attributes. Tukey’s b multiple range test was used for means comparison. Statistical significance was assessed at *p* < 0.05.

## 3. Results and Discussion

### 3.1. Headspace Gas Analysis

**Figure 2:**
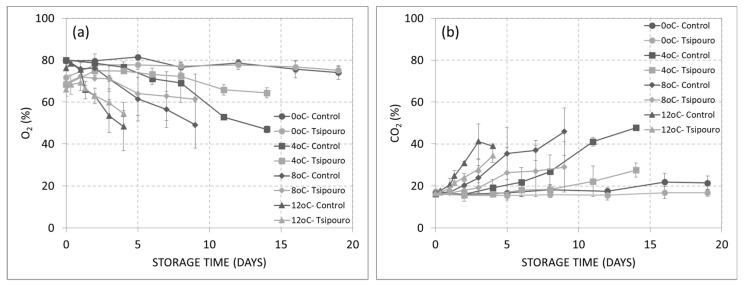
Evolution of (**a**) O_2_% and (**b**) CO_2_% on ground beef (500 g) packaged with absorbent pads supplemented with a total volume of 50 mL “*tsipouro*” (40 mL under the ground beef piece and 10 mL under the labelling, facing the headspace) and stored under 80% O_2_: 20% CO_2_ at 0, 4, 8, and 12 °C. Untreated samples served as controls.

**Figure 3:**
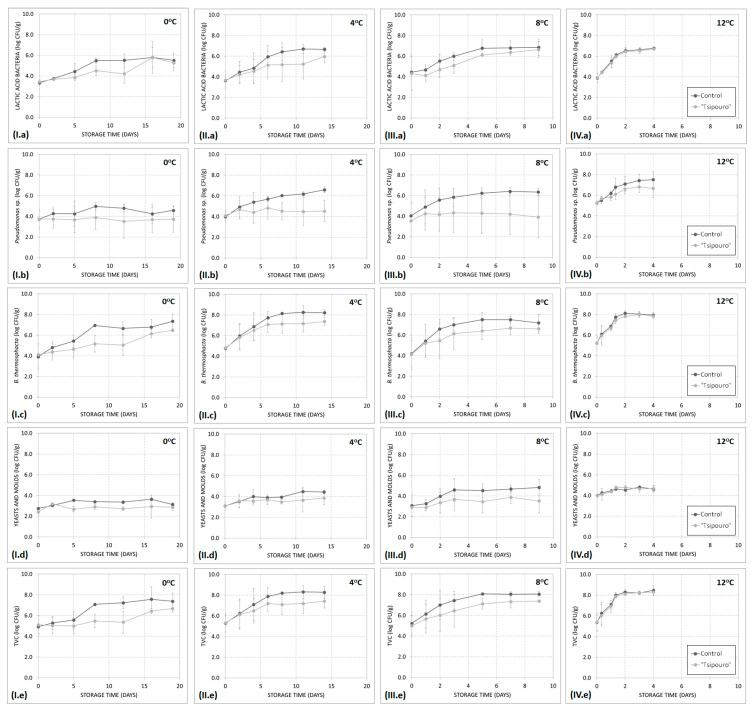
Growth curves of (**a**) LAB, (**b**) pseudomonads, (**c**) *B. thermosphacta*, (**d**) yeasts and moulds, and (**e**) TVC in ground beef (500 g) packaged with absorbent pads supplemented with a total volume of 50 mL “*tsipouro*” (40 mL under the ground beef piece and 10 mL under the labelling, facing the headspace) under 80% O_2_: 20% CO_2_ during isothermal storage at (**I**) 0 °C, (**II**) 4 °C, (**III**) 8 °C, and (**IV**) 12 °C. Untreated samples served as controls.

Oxygen decrease occurred with concomitant carbon dioxide evolution during storage at all temperatures except for 0 °C (Figure 2a,b). Gases variations took place faster as storage temperature was increased from 4 to 12 °C, regardless of treatment. At 0 °C, gases retained close to the initial MAP composition both on ground beef treated with “*tsipouro*” and controls for almost 20 days in accordance with the recorded low TVC growth (Figure 2a,b and Figure 3I.e). Controls stored at temperatures > 4 °C displayed a continuous change in headspace gas composition from the beginning of storage. On the contrary, in samples treated with “*tsipouro*”, O_2_% and CO_2_% remained stable for 8, 3, and 1.3 days during storage at 4, 8, and 12 °C, respectively, followed by a linear change on both gases. At each of the aforementioned critical time points per temperature, TVC have reached almost 7.0 log CFU/g, suggesting that headspace gas composition could be used as a spoilage indicator (Figure 3II.e–IV.e). With regards to controls, similar trends were found with previous studies on beef steaks packaged in high oxygen MAP and the storage of 21 days at 4 °C resulted in a modified atmosphere of ca. 50% O_2_ and 50% CO_2_ [34]. Notably, on day 0, the concentration (%) of O_2_ in packages of ground beef packaged with “*tsipouro*” ranged from 63.7 to 71.8% instead of the target MAP composition of ca. 80%, achieved in controls (Figure 2a). The latter observation may be related to the rapid release of “*tsipouro*” vapors right after its addition in the absorbent pads, which possibly increased vapor pressure in the package headspace and thus, partly limited the filling of packaging with the targeted MAP composition. However, even in this case, “*tsipouro*” showed higher stability on O_2_ levels compared to controls, regardless of storage temperature, suggesting a strong preservation potential.

### 3.2. Kinetics of Microbiological Data and Model Development/Validation

The applied volume of “*tsipouro*” (40 mL) on the absorbent pad placed underneath the ground beef was decided after conducting preliminary experiments, following similar set up to the main experiment (namely 500 g ground beef, MAP, TVC, and colour measurements) (see Section 2.2) and testing “*tsipouro*” volumes of 20, 30, 40, and 60 mL combined with storage at 7 °C. Growth of TVC was delayed by increasing the applied volume of “*tsipouro*”, resulting in 0 (20 mL), 2 (30 mL), and 5 days (40 mL and 60 mL) shelf-life extension (data not shown). However, the volume of 40 mL was eventually selected for the main experiment, because ground beef samples packaged with this volume showed more “bloomed” red colour compared to samples packaged with 60 mL “*tsipouro*”.

The changes in relative levels of spoilage association of ground beef packaged with absorbent pads supplemented with “*tsipouro*” or not (controls), were dependent (*p* < 0.05) on storage temperature (Figure 3). This is also supported by the maximum specific growth rates (*µ_max_*), which significantly increased (*p* < 0.05) with temperature from 0 to 12 °C, regardless of treatment and microorganism (Table 1). Higher temperatures also increased (*p* < 0.05) the maximum population density (*N_max_*) of *B. thermosphacta,* but this was counteracted by “*tsipouro*”, resulting in maximum population levels from 6.38 ± 0.45 to 7.93 ± 0.20 log CFU/g (Table 1). *B. thermosphacta* dominated the microbial association of ground beef, while LAB were second in dominance, revealing a high growth potential at all assays (Figure 3). The dominance of those two, facultative anaerobic (*B. thermosphacta*) and microaerophilic (LAB), microorganisms is mainly associated with the type of MAP applied. Previous studies have reported *B. thermosphacta* and/or LAB as the most prevalent in meat products such as pork steaks, beef patties, and beef steaks packaged under high oxygen MAP, namely 60‒80% O_2_: 40‒20% CO_2_ [15,34,35]. Yeasts‒moulds had limited contribution to the spoilage association, profoundly due to their aerobic metabolism, which is not favoured by elevated CO_2_ and/or O_2_ levels compared to air (Figure 3I.d,IV.d). Specifically, they showed either 100% inhibition during storage at 0 °C or an average increase of *max*. 2.0 log CFU/g when samples were stored at temperatures higher than 4 °C, regardless of treatment. Although *Pseudomonas* species are recognized as obligate aerobes with solely aerobic respiratory metabolism [36], a moderate growth rate was observed on controls reaching population of 6.0–7.0 log CFU/g at the end of storage above 4 °C where the recorded oxygen was close to 50% (Figure 2 and Figure 3II.b–IV.b). Similarly, Hilgarth et al., (2017) detected *Pseudomonas* spp. in early, mid and very late spoilage stage on high oxygen MAP beef steaks stored at 10 °C even with up to 90% CO_2_ and oxygen level down to 1% [34]. Moreover, it is also notable that when “*tsipouro*” was applied on ground beef, standard deviations of the enumerated pseudomonads populations were significantly high in contrast to the respective on controls. From the antimicrobial treatment standpoint, which is the main objective of the present study, growth rates of *B. thermosphacta* and LAB were significantly (*p* < 0.05) delayed by the presence of “*tsipouro*” compared to controls, at the majority of storage temperatures. In fact, for *B. thermosphacta*, ground beef packaged with “*tsipouro*” showed *µ_max_* ca. 2-fold lower than the controls at 0–8 °C (*p* < 0.05) (Table 1). In previous study of our research group, vapours of “tsipouro” was among the most effective antimicrobials along with “*ouzo*”, and “*raki*” doubling the shelf-life of pork meat packaged under at 4 °C [15]. Regarding the pH, changes in controls (from 5.54 to 5.89) and in ground beef packaged with “*tsipouro*” (from 5.61 to 5.83) were non-significant (*p* ≥ 0.05), at all storage temperatures (data not shown). Overall, the antimicrobial active packaging with “*tsipouro*” significantly extended shelf-life of packaged ground beef as calculated by *B. thermosphacta* growth (specific spoilage organism) and Equation (1). Specifically, shelf-life of controls versus “*tsipouro*” was estimate from 28.0 ± 3.8 to 49.3 ± 1.7 days at 0 °C, from 9.5 ± 3.5 to 14.2 ± 6.8 days at 4 °C and from 5.1 ± 0.9 to 10.1 ± 4.0 days at 8 °C (*p* < 0.05), while the shelf-life at 12 °C remained unaffected (*p* ≥ 0.05).

With regards to secondary modelling, *µ_max_* of dominant spoilage bacteria of ground beef packaged with or without “*tsipouro*” was described as a function of storage temperature by square root and Arrhenius models, while the estimated parameters and goodness-of-fit statistics are shown in Table 2. The average estimated activation energies of Arrhenius model for *μ_max_* of *B. thermosphacta* and LAB in controls were 90.2 ± 12.2 kJ/mol and 92.8 ± 10.4 kJ/mol, respectively, while in ground beef packaged with “*tsipouro*” equaled to 87.2 ± 17.1 kJ/mol (*B. thermosphacta*) and 124.8 ± 15.0 kJ/mol (LAB) (Table 2). The recorded values of *E_a_* in controls were close to the ones reported in previous studies. Specifically, the reported range of *E_a_* ranged from 73.2 to 91.9 kJ/mol for *B. thermosphacta*, while from 92.8 to 99.6 kJ/mol for LAB during storage of ground beef and/or pork under MAP or aerobic conditions [37,38,39].

**Figure 4:**
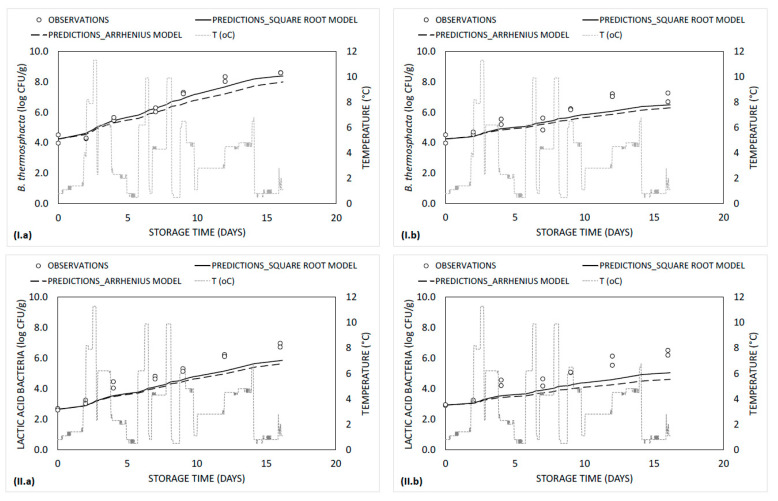
Simulated growth curves and observed data of (**I**) *B. thermosphacta* and (**II**) LAB in ground beef (500 g) packaged (**a**) without (controls) or (**b**) with absorbent pads supplemented with a total volume of 50 mL “*tsipouro*” (40 mL under the ground beef piece and 10 mL under the labelling, facing the headspace) under 80% O_2_: 20% CO_2_ and stored at dynamic temperature conditions.

Goodness-of-fit indices (R^2^ and RMSE) indicated that both secondary models adequately described the microbial growth data (Table 2). A good agreement between fitted and observed values of *B. thermosphacta* on controls and “*tsipouro*” was observed, since A*f* and B*f* were close to 1, both with square root model (A*f*: 1.05–1.09; B*f*: 0.94–0.99) or Arrhenius equation (A*f*: 1.07–1. 11; B*f*: 0.92–0.96) [29] (Figure 4I.a,I.b). The latter is also enforced by the fact that the pRE indices on controls (1.000) and “*tsipouro*” samples (1.000), met the criterion of pRE ≥ 0.70 for both models tested [32]. In case of LAB, both models tend to constantly (but not substantially) underestimate their growth, thus resulting in pRE ranging from 0.714 to 1.000 regardless of model tested (Figure 4II.a,II.b). Overall, comparing the two models studied, square root model showed slightly better performance compared to Arrhenius, regardless of microorganism and treatment.

### 3.3. Colour Measurements and Model Development/Validation of a* Value

**Figure 5:**
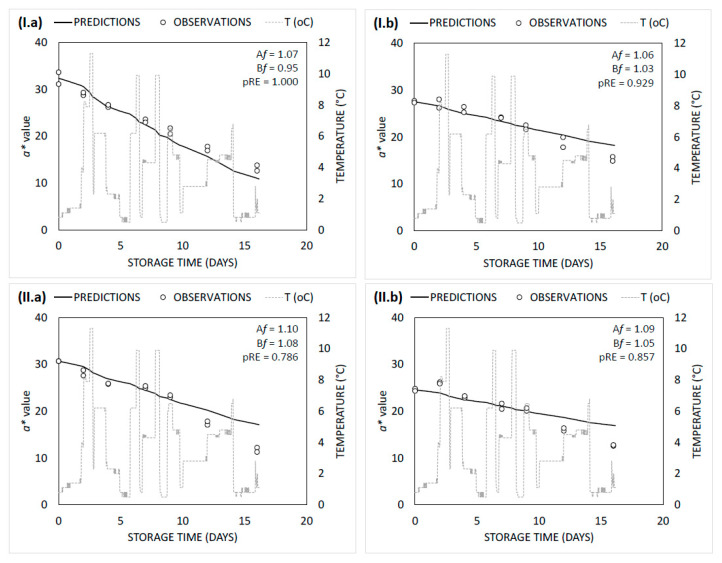
Simulated lines and observed data of a* value on the (**I**) surface and (**II**) bottom side of ground beef (500 g) packaged (**a**) without (controls) or (**b**) with absorbent pads supplemented with a total volume of 50 mL “*tsipouro*” (40 mL under the ground beef piece and 10 mL under the labelling, facing the headspace) under 80% O_2_: 20% CO_2_ and stored at dynamic temperature conditions.

Vivid red colour of ground beef is a primary attribute that consumers set as a key for quality acceptance on purchase. In fact, the presence of any discoloration, or metmyoglobin affects consumer preferences and may result in a total loss to retail stores of over $1 billion dollars annually [40]. Thus, colour evaluation becomes an important factor for the meat industry not only for quality control, but also for the commercialization of a product [6,41]. Considering the above, colour evaluation was included in the present study by monitoring L*, a*, and b* parameters on the surface and the bottom of ground beef packaged with or without “*tsipouro*”. Specifically, L* values remained unchanged (*p* ≥ 0.05), while b* decreased (*p* < 0.05) during storage, at all temperatures, sampling sides (i.e., top surface or bottom) and treatment (data not shown). Notably, on day 0, the recorded a* values were lower (24.4–28.6) in ground beef samples treated with “*tsipouro*” compared to controls (28.5–32.0), suggesting an acute effect of the distillate on the red colour of the product (Figure 5). Moreover, samples treated with “*tsipouro*” showed greater stability of a* value during storage compared to controls. The latter observation is also reflected on the estimated *k_colour_* per treatment (*p* < 0.05) (Table 3). A possible interpretation may be the antioxidant properties of “*tsipouro*” due to the presence of phenolic compounds in its composition, combined with the well-known property of high oxygen MAP for maintenance of the myoglobin pigment in its oxygenated form, oxymyoglobin, inhibiting the rapid formation of metmyoglobin, and thus, enhancing red colour [42,43]. Moreover, the recorded coloured stability in samples packaged with “*tsipouro*” was even higher at the bottom of the ground piece compared to surface. This is possible related to the direct contact of the bottom side of meat with “*tsipouro*”, contrary to the surface, where any antioxidant activity was indirectly conveyed through vapours.

Thus, a* which corresponds to red colour, was selected as the critical one for modelling purposes among the three parameters. Changes in a* value with time was mathematically described by a linear equation (Equation (3)). Controls (0.846–0.976) and “*tsipouro*” samples (0.769–0.961) showed good R^2^, indicating that this linear equation sufficiently described the effect of storage temperature on a* (Table 4). The estimated parameters (*E_a_* and *k_ref_*) and performance statistics of the Arrhenius model for a* value are illustrated in Table 3. In controls, the calculated activation energies of a* value on the surface and bottom side were 99.2 ± 11.3 kJ/mol and 89.0 ± 14.1 kJ/mol, respectively, while in ground beef packaged with “*tsipouro*” values of 63.6 ± 10.6 kJ/mol (*surface*) and 86.1 ± 8.0 kJ/mol (bottom) were estimated. R^2^ (0.870–0.950) and RMSE (0.025–0.076) indicated that the secondary models adequately described the data. Regarding the performance of the a* value secondary models under dynamic temperature conditions, predictions and observations showed good agreement for both sides of ground beef packaged either with “*tsipouro*” or not (controls), since A*f* and B*f* ranged from 1.06 to 1.10 and 0.95 to 1.08, respectively. Moreover, pRE indices were ranging from 0.786 to 1.000, meeting the criterion of of pRE ≥ 0.70 [32] and indicating that these models could be a reliable mathematical tool for predicting red colour variation not only in controls but also in case a ground beef industry decides to adopt the proposed antimicrobial active packaging with “*tsipouro*” (Figure 5).

### 3.4. Sensory Evaluation

**Figure 6:**
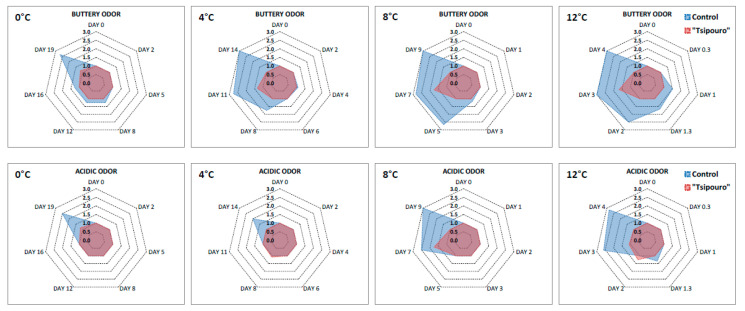
Mean results of buttery and acidic odour of ground beef packaged with absorbent pads supplemented with a total volume of 50 mL “*tsipouro*” (40 mL under the ground beef piece and 10 mL under the labelling, facing the headspace) under 80% O_2_: 20% CO_2_ during isothermal storage at 0, 4, 8, and 12 °C. Untreated samples served as controls. The hedonic scale was set from 1 to 3 with 0.5 intervals. Score of 2 was the rejection threshold.

Analysis of variance performed using the sensory attribute scores was indicative of significant differences (*p* < 0.05). It is well-known that *B. thermosphacta* may cause creamy buttery off-odours probably due to the production of acetoin from glucose, while acidic off-odours may be detected when LAB prevail [33]. In general, ground beef samples packaged with “*tsipouro*” showed significant stability (*p* < 0.05) compared to controls during storage at all temperatures (Figure 6). Specifically for buttery off-odour, controls were evaluated by the panellists with high rejection scores (≥2) after 19, 11, 5, and 2 days during storage at 0, 4, 8, and 12 °C, respectively (Figure 6). At each of the aforementioned critical time points per temperature, *B. thermosphacta* have almost reached 8.0 log CFU/g [44] (Figure 3I.c–IV.c). On the contrary, ground beef packaged with “*tsipouro*” pads received markedly lower scores against the above rejection criterion (i.e., <2), at all storage temperatures and both studied off-odours.

With regards to the perception of ethanol odour, the majority of panellists noticed that ethanol was easily detectable until the middle of the storage, depending on the temperature (data not shown). It was reported more explicitly at lower temperatures, namely at 0 and 4 °C. According to panellists’ comments, when ethanol was not detected, buttery or acidic odour dominated, possibly overlapping ethanol aroma. However, even in cases where ethanol was clearly perceived, it was characterized as a quite pleasant, “cool” odour.

### 3.5. SPME/GC-Flame Ionization Detector Analysis

In the present section, the relative differences of ethanol in ground beef when “*tsipouro*” was applied compared to controls over storage time were monitored. Considering that, in the developed antimicrobial active packaging based on “*tsipouro*”, ethanol would migrate to ground beef through two mechanisms, diffusion and evaporation, 2 types of sampling took place, i.e., from the surface and the bottom side of each ground beef piece (Section 2.12).

**Figure 7:**
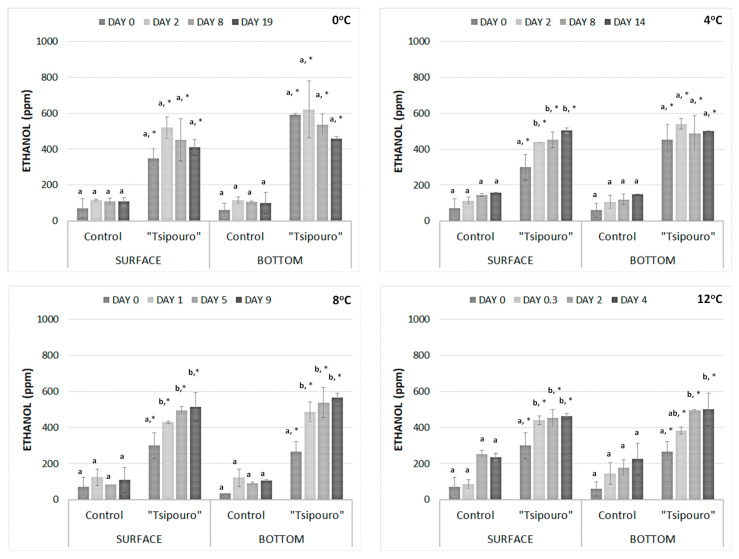
Ethanol in ground beef (ppm) packaged with absorbent pads supplemented with a total volume of 50 mL “*tsipouro*” (40 mL under the ground beef piece and 10 mL under the labelling, facing the headspace) under 80% O_2_: 20% CO_2_ at 0, 4, 8, and 12 °C. Samplings were performed in depth of 1 cm either from the surface or from the bottom side of the 500 g ground beef piece. Untreated samples served as controls. Bars under the same conditions (treatment, sampling side, and storage temperature) having different lowercase letter are significantly different from each other (*p* < 0.05); Star indicates statistical significance of “*tsipouro*” treatment in comparison to controls under the same conditions (sampling side, storage day, and temperature) (*: *p* < 0.05).

With regards to GC data, the detected ethanol in samples packaged with “*tsipouro*” was ca. 3 to 6‒fold higher than the respective values of ethanol in controls (*p* < 0.05), regardless of temperature, storage day, and sampling side (Figure 7). In controls, the detected ethanol was likely the result of microbial metabolic activity. No significant differences (*p* ≥ 0.05) in ethanol content were observed between samples from the surface and bottom of ground beef, refuting the potential hypothesis that migration through diffusion could reveal higher ethanol content compared to evaporation, due to the direct contact between the antimicrobial (on the pad) and ground beef. On the contrary, the results potentially indicated that recorded ethanol during storage may be the combined result of both migration mechanisms, leading eventually to a gradual ethanol equilibrium in the packaging headspace during storage (Figure 7). On samples packaged with “*tsipouro*” pads, the latter equilibrium took place earlier as the storage temperature increased from 0 to 12 °C, since the evaporation rate of ethanol increased. specifically, the ethanol equilibrium was recorded close to the suggested chilling temperatures (0 °C and/or 4 °C), in ground beef samples either from the top surface or the bottom, while an initial increase was rapidly followed by equilibrium close to abuse temperatures (8 and 12 °C). However, it should be also taken into account that the suggested ground beef with “*tsipouro*” is a product that will be consumed after thermal treatment, thus any residual perception of ethanol through odour or via taste after cooking would be negligible.

## 4. Conclusions

It is well-known that during a new product development and prior to its commercialization, technological concerns related to its shelf-life and sensory properties along with its implementation in industrial scale may need to be addressed. The present study tried to systematically deal with the aforementioned concerns. Specifically, the results showed that the developed antimicrobial active packaging based on “*tsipouro*” combined with high oxygen MAP significantly extended (*p* < 0.05) the shelf-life of a perishable meat products like ground beef (ca. 2-fold compared to controls) during storage at chill (at 0 and 4 °C) or abuse temperatures (at 8 °C). Moreover, the transfer of such innovative applications in large scale is a common issue for the industry, since modification and/or purchase of new equipment may be needed. Considering that, the developed product-packaging of the present study was deliberately based on the commercial packaging of ground beef, while the applied modifications took place using materials (absorbent pads) already existing in the package. Thus, in case a meat industry decides to launch such an antimicrobial active packaging, the additional cost would be limited in the applied antimicrobial and minor potential modifications across the industrial line. Nonetheless, cost and market analyses are also necessary for tailor-made evaluation of whether the proposed antimicrobial active packaging technology is feasible by the industry of concern and thus, whether it may be readily channelled to the market.

## Figures and Tables

**Table 1:** Kinetic parameters and statistics of *B. thermosphacta* and LAB estimated from the primary model of Baranyi and Roberts on ground beef (500 g) packaged with absorbent pads supplemented with 50 mL of “*tsipouro*” (40 mL under the ground beef piece and 10 mL under the labelling, facing the headspace) and stored at 0, 4, 8, and 12 °C under 80% O_2_: 20% CO_2_. Untreated samples served as controls.

Microorganism/ Treatment	Temperature (°C)	*Ν*_0_ (log CFU/g) ^1^	*Ν_max_* (log CFU/g) ^1^	*µ_max_* (day^−1^) ^1^	*λ* (days)
***B. thermosphacta***					
Control	0	3.91 ± 0.96 ^A^	7.00 ± 0.56 ^A^	0.36 ± 0.14 ^A^	N.A. ^2^
	4	4.75 ± 0.75 ^AB^	8.32 ± 0.31 ^C^	0.65 ± 0.18 ^B^	N.A.
	8	4.18 ± 1.27 ^AB^	7.55 ± 0.34 ^AB^	1.26 ± 0.06 ^C^	N.A.
	12	5.82 ± 0.21 ^B^	8.04 ± 0.24 ^BC^	1.46 ± 0.06 ^C^	N.A.
“*Tsipouro*”	0	4.10 ± 0.89 ^A^	6.38 ± 0.45 ^A^	0.14 ± 0.04 ^A^*	N.A.
	4	5.06 ± 1.09 ^A^	7.27 ± 0.55 ^AB^*	0.37 ± 0.16 ^A^*	N.A.
	8	4.14 ± 1.08 ^A^	6.66 ± 0.50 ^A^*	0.72 ± 0.26 ^B^*	N.A.
	12	5.85 ± 0.17 ^A^	7.93 ± 0.20 ^B^	1.15 ± 0.11 ^C^	N.A.
**LAB**					
Control	0	2.92 ± 0.31 ^A^	6.51 ± 0.39 ^A^	0.28 ± 0.02 ^A^	N.A.
	4	3.62 ± 0.53 ^AB^	6.55 ± 0.14 ^A^	0.45 ± 0.19 ^A^	N.A.
	8	4.44 ± 1.47 ^B^	6.85 ± 0.65 ^A^	0.87 ± 0.04 ^B^	N.A.
	12	3.89 ± 0.34 ^AB^	6.68 ± 0.06 ^A^	1.64 ± 0.07 ^C^	N.A.
“*Tsipouro*”	0	3.11 ± 0.21 ^A^	6.74 ± 0.77 ^A^	0.23 ± 0.05 ^A^	N.A.
	4	3.90 ± 0.95 ^A^	6.13 ± 1.11 ^A^	0.17 ± 0.06 ^A^*	N.A.
	8	4.31 ± 1.36 ^A^	6.75 ± 0.67 ^A^	0.65 ± 0.16 ^B^*	N.A.
	12	3.92 ± 0.35 ^A^	6.61 ± 0.19 ^A^	1.46 ± 0.06 ^C^*	N.A.

^1^ Mean value ± standard deviation (*n* = 4); ^2^ N.A.: Non Applicable; *N*_0_, *Ν_max_* and *µ_max_* values within the same treatment and microorganism having different uppercase letter are significantly different from each other (*p* < 0.05); Star indicates statistical significance of “*tsipouro*” treatment in comparison to controls under the same conditions (microorganism and storage temperature) (*: *p* < 0.05).

**Table 2:** Estimated parameters and statistics of the developed secondary square root growth models and Arrhenius models of *B. thermosphacta* and LAB on ground beef (500 g) packaged with absorbent cloths supplemented with 50 mL of “*tsipouro*” (40 mL under the ground beef piece and 10 mL under the labelling, facing the headspace) and stored at 0, 4, 8, and 12 °C under 80% O_2_: 20% CO_2_. Untreated samples served as controls.

Parameters and Statistical Indices	*B. thermosphacta*				LAB			
	Control		“*Tsipouro*”		Control		“*Tsipouro*”	
	Estimate	*p*-Value	Estimate	*p*-Value	Estimate	*p*-Value	Estimate	*p*-Value
**Square Root Model**								
*b*	0.054 ± 0.005	0.000	0.059 ± 0.006	0.000	0.059 ± 0.008	0.000	0.063 ± 0.009	0.000
*T*_0_ (°C)	−11.3 ± 1.6	0.000	−6.2 ± 1.2	0.000	−7.8 ± 1.3	0.000	−4.9 ± 1.6	0.000
R^2^	0.865		0.782		0.805		0.760	
RMSE	0.017		0.016		0.019		0.029	
**Arrhenius Model**								
*E_a_* (kJ/mol)	90.2 ± 12.2	0.000	87.2 ± 17.1	0.002	92.8 ± 10.4	0.000	124.8 ± 15.0	0.000
ln *k_ref_* (day^−1^)	−0.586 ± 0.092	0.001	−1.167 ± 0.129	0.000	−0.833 ± 0.081	0.000	−1.448 ± 0.113	0.000
R^2^	0.901		0.813		0.859		0.831	
RMSE	0.057		0.111		0.082		0.173	

**Table 3:** Estimated deterioration rate of a* value (*k_colour_* (day^−1^)) and statistics on the surface and bottom side of ground beef (500 g) packaged with absorbent pads supplemented with 50 mL of “*tsipouro*” (40 mL under the ground beef piece and 10 mL under the labelling, facing the headspace) and stored at 0, 4, 8, and 12 °C under 80% O_2_: 20% CO_2_. Untreated samples served as controls.

Sampling Side	Treatment	Temperature (°C)	*K_colour_* (day^−1^)	R^2^
Surface	Control	0	−0.852 ± 0.095 ^A^	0.976
		4	−1.079 ± 0.028 ^A^	0.949
		8	−2.060 ± 0.093 ^B^	0.937
		12	−5.322 ± 0.032 ^C^	0.971
	“*Tsipouro*”	0	−0.343 ± 0.004 ^A^*	0.769
		4	−0.737 ± 0.057 ^AB^*	0.961
		8	−0.838 ± 0.047 ^AB^*	0.888
		12	−0.953 ± 0.284 ^B^*	0.887
Bottom	Control	0	−0.472 ± 0.074 ^A^	0.846
		4	−1.027 ± 0.021 ^A^	0.972
		8	−1.039 ± 0.153 ^A^	0.869
		12	−2.966 ± 0.532 ^B^	0.906
	“*Tsipouro*”	0	−0.242 ± 0.031 ^A^*	0.775
		4	−0.578 ± 0.028 ^B^*	0.901
		8	−0.823 ± 0.045 ^C^*	0.913
		12	−1.261 ± 0.088 ^D^*	0.837

*k_colour_* values within the same treatment and sampling side having different uppercase letter are significantly different from each other (*p* < 0.05); Star indicates statistical significance of “*tsipouro*” treatment in comparison to controls under the same conditions (sampling side and storage temperature) (*: *p* < 0.05).

**Table 4:** Estimated parameters and statistics of the developed secondary Arrhenius models of a* value on the surface and bottom side of ground beef (500 g) packaged with absorbent pads supplemented with 50 mL of “*tsipouro*” (40 mL under the ground beef piece and 10 mL under the labelling, facing the headspace) and stored at 0, 4, 8, and 12 °C under 80% O_2_: 20% CO_2_. Untreated samples served as controls.

Parameters and Statistical Indices	Control		“*Tsipouro*”	
	Estimated Value	*p*-Value	Estimated Value	*p*-Value
**Surface**				
*E_a_* (kJ/mol)	99.2 ± 11.3	0.000	63.6 ± 10.6	0.002
ln *k_ref_* (day^−1^)	0.279 ± 0.086	0.017	−0.532 ± 0.070	0.001
R^2^	0.927		0.878	
RMSE	0.049		0.032	
**Bottom**				
*E_a_* (kJ/mol)	89.0 ± 14.1	0.001	86.1 ± 8.0	0.000
ln *k_ref_* (day^−1^)	−0.172 ± 0.106	0.156	−0.743 ± 0.061	0.000
R^2^	0.870		0.950	
RMSE	0.076		0.025	
